# The Impact of Coronary Sinus Reducer on Arrhythmic Properties in Patients with Refractory Angina

**DOI:** 10.31083/j.rcm2412368

**Published:** 2023-12-26

**Authors:** Miha Mrak, Tadej Žlahtič, Vito Starc, Maja Ivanovski, Matjaž Bunc, David Žižek

**Affiliations:** ^1^Department of Cardiology, University Medical Centre Ljubljana, 1000 Ljubljana, Slovenia; ^2^Faculty of Medicine, University of Ljubljana, 1000 Ljubljana, Slovenia; ^3^Institute of Physiology, Faculty of Medicine, University of Ljubljana, 1000 Ljubljana, Slovenia

**Keywords:** electrocardiogram, refractory angina, arrhythmia, coronary sinus reducer

## Abstract

**Background:**

Treatment with a coronary sinus reducer (CSR) is a new therapeutic option for refractory angina patients. Preclinical studies have shown antiarrhythmic properties of coronary sinus narrowing. The possible antiarrhythmic effect of CSR implantation is unknown. This study aimed to determine the possible antiarrhythmic effects of CSR implantation as assessed by high-resolution electrocardiogram (hrECG) parameters.

**Methods:**

24 patients from the Crossroad study randomized to either CSR treatment (n = 12) or a sham procedure (n = 12) had hrECG recorded at baseline and after 6 months. T-peak and T-end interval (TpTe) defined as the time difference between the peak amplitude of the T wave and the global end of the T wave, spatial angle between QRS complex and T axis defined as the angle between the ventricular depolarization and repolarization vectors using maximal (QRSTP) and mean (QRSTM) vector amplitudes and spatial ventricular gradient (SVG) calculated as integral of ECG voltages over the entire QRST complex were analyzed. Additionally, we analyzed parameters of QT and heart rate variability using time and frequency domain.

**Results:**

At baseline, all analyzed parameters were comparable between both groups and heart rate remained constant. The intragroup analysis did not show any significant change in TpTe, QRSTP, QRSTM, SVG, QT, and heart rate variability at follow-up. Furthermore, intergroup comparison between CSR implantation and sham procedure also did not show any significant difference in the change of analyzed parameters.

**Conclusions:**

Compared to the sham procedure, CSR implantation did not demonstrate a significant impact on the arrhythmogenic substrate assessed with hrECG.

**Clinical Trial Registration:**

Unique Identifier: NCT04121845, https://classic.clinicaltrials.gov/ct2/show/NCT04121845.

## 1. Introduction

Treatment with a coronary sinus reducer (CSR) is a new therapeutic option for refractory angina patients. It is an hourglass-shaped stainless steel mesh with a central narrowing implanted in the distal coronary sinus. After endothelization, it creates a focal narrowing of the coronary sinus lumen to approximately 3 mm, leading to increased venous pressure in the proximal coronary sinus [1, 2]. The increased pressure gradient is transmitted backwards to the myocardial microcirculation, improving the perfusion ratio between the ischemic subendocardium and the non-ischemic subepicardium [3, 4].

As CSR does affect myocardial perfusion, increased capillary hydrostatic pressure, and myocardial blood flow, this therapy may also potentially impact the arrhythmic properties of the ischemic myocardium. While there is no clear long-term clinical evidence of its arrhythmic effects, the preclinical trials, especially in the acute setting, showed a favorable association between coronary sinus narrowing and the inducibility of ventricular fibrillation in both ischemic and non-ischemic hearts [5, 6].

This study aimed to evaluate the possible impact of CSR implantation on the arrhythmogenic substrate in patients with refractory angina pectoris and evidence of reversible ischemia.

## 2. Materials and Methods

### 2.1 Patient Selection

The coronary sinus reducer implantation for ischemia reduction (CrossRoad) study (ClinicalTrials.gov identifier NCT04121845) was a randomized, single-center, double-blind, sham-controlled study that enrolled eligible patients who underwent treatment with CSR at UMC Ljubljana between 1st January 2019 and 31st December 2021. All patients had symptomatic angina for more than 3 months and were classified in class II–IV according to the Canadian Cardiovascular Society (CCS). Patients had to be treated with optimal medical therapy for at least one month and had reversible ischemia in the anterior, lateral, and inferolateral left ventricular walls confirmed by single photon emission tomography (SPECT). Patients with unstable angina within the last 30 days, acute myocardial infarction within the last 90 days, recent successful revascularization, decompensated heart failure, and severe valvular heart disease were excluded from the study. The study was approved by the national ethics committee. Before the inclusion, all patients signed the informed written consent to participate in the study.

### 2.2 Procedures

**Fig. 1.**
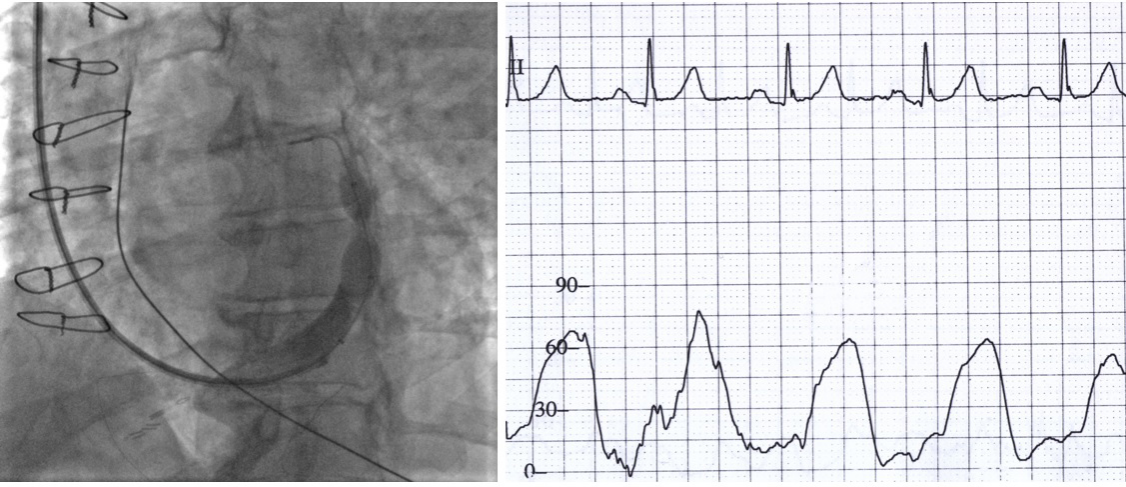
**Coronary sinus venography and occlusion pressure measurement**. Left: Venography during coronary sinus reducer (CSR) implantation, showing the final position of the CSR in the distal coronary sinus. The CSR catheter balloon is still inflated. Right: Pressure tracing in the proximal coronary sinus during the balloon inflation. Systolic pressure was measured during this phase of the procedure to reduce heterogeneity associated with an extensive network of Thebesian veins.

Patients were randomized to either CSR implantation or a sham procedure. The CSR implantation technique is already described elsewhere [7, 8]. Following the right internal jugular vein puncture, the right atrial pressure was measured, followed by cannulation and venography of the coronary sinus. CSR was implanted in the distal coronary sinus with special care not to obstruct any greater tributary and with 10% oversizing to ensure stability. During CSR balloon inflation, occlusion pressure of the coronary sinus proximal to the CSR was measured. After final CSR positioning in the distal coronary sinus, the CSR balloon catheter was connected to the pressure transducer via incompressible plastic tubing. The pressure transducer was positioned at the level of the phlebostatic axis, and the reference point was set to the atmospheric pressure. During the inflation of the CSR balloon, the pressure waveform was recorded. The occlusion pressure was determined as a peak pressure recorded during systole (Fig. 1). Final venography confirmed the appropriate position of the device. A sham procedure was performed by the same experienced operator and in the same catheterization laboratory. Cannulation of the right internal jugular vein was followed by right atrial pressure measurement. The time of the procedure was similar to the CSR implantation. Both procedures were performed in hearing isolation. All medical personnel, apart from those performing the procedure, were blinded to the patient allocation.

### 2.3 ECG Data and Measures of Repolarization Heterogeneity

Five-minute 12-lead high-resolution electrocardiogram (hrECG) (Cardiax computer ECG, IMED KFT., Budapest, Hungary) with a sampling rate of 1 kHz and 300 Hz low pass filter was recorded at baseline and after 6 months. The researcher who analyzed hrECG data was unaware of the patient allocation. Premature ventricular complexes and complexes with severe artifacts were removed from further analysis. The remaining QRS-T complexes were averaged to calculate median beats. Orthogonal leads were calculated using the Kors regression transformation method. T-peak and T-end interval (TpTe) was measured from vector signal and was defined as the time difference between the T wave’s peak amplitude and the T wave’s global end. The spatial angle between the QRS and T axis (QRS-T angle) was defined as the angle between the ventricular depolarization (QRS) and repolarization (T wave) vectors using maximal amplitudes of the QRS and T vectors, reported as QRSTP angle, and using mean amplitudes of the QRS and T vectors reported as QRSTM angle. QT interval for QT variability (QTV) calculations was normalized for heart rate ($\frac{d\,Q\,T}{d\,R\,R}$) and measured from the constructed vector signal. QTV was reported as the QT variability index:

$Q\,T\,V\,i=l\,o\,g\,\frac{Q\,T\,V\,N}{H\,R\,V\,N}$, where $Q\,T\,V\,N=\frac{S\,D\,Q\,T^{2}}{Q\,T\,m\,e\,a\,n^{2}}$ and $H\,R\,V\,N=\frac{H\,R\,V\,A\,R}{H\,R\,m\,e\,a\,n^{2}}$ and as QT variability ratio:

$$V\,R=\frac{S\,T\,V\,Q\,T}{S\,T\,V\,R\,R},w\,h\,e\,r\,e\,S\,T\,V\,Q\,T=\sum \frac{\left|Q\,T\,n+1-Q\,T\right|}{N\,\sqrt{2}}$$

[9].

Spatial ventricular gradient (SVG) amplitude was calculated as an integral of ECG voltages over the entire QRS-T complex:

$$SVG=\left[\int_{Q\,b\,e\,g}^{T\,e\,n\,d}Vx\left(t\right)dt,\int_{Q\,b\,e\,g}^{T\,e\,n\,d}Vy\left(t\right)dt,\int_{Q\,b\,e\,g}^{T\,e\,n\,d}Vz\left(t\right)dt,\right]$$

obtained from all three axes of orthogonal ECG [10]. Heart rate variability parameters were calculated with power spectral density analysis using a Lomb-Scargle periodogram [11]. High, low, and total frequency powers are reported. As a measure of heart rate variability, we also included a time domain parameter reported as the standard deviation of the normal-to-normal intervals (SDNN).

### 2.4 Randomization and Statistical Analysis

Randomization was performed using block randomization with a block size of 4 generated by the online statistical software (http://www.jerrydallal.com/random/randomize.htm, visited on 6th May 2018). Categorical variables are presented as frequencies and percentages, and continuous variables as mean ± standard deviation (SD) or median and interquartile range. Categorical variables were compared using Chi-square or Fisher exact tests. The normality of distribution for continuous variables was evaluated by the Kolmogorov-Smirnov test. Intra- and intergroup comparisons were performed using paired or independent sample *t*-test, Wilcoxon signed-rank, and rank-sum test as appropriate. Statistical analysis was performed in SPSS Statistics, version 22.0 (IBM Corp., Armonk, NY, USA). A two-sided *p*-value of 0.05 was considered statistically significant.

## 3. Results

Of the 25 patients enrolled in the Crossroad study, 24 were included in the hrECG analysis. One patient was excluded due to their permanent pacemaker rhythm. Twelve patients received CSR and 12 patients underwent a sham procedure. Baseline characteristics are shown in Table 1 and did not differ between both groups.

**Table 1.** **Baseline characteristics**.

		CSR (n = 12)	Sham (n = 12)	*p*
Age–years ± SD		69.8 ± 10.5	69.8 ± 11.7	0.92
Male, n (%)		10 (83.3%)	10 (83.3%)	1.00
Diabetes		3 (25%)	4 (33.3%)	1.00
Prior PCI, n (%)		6 (50.0%)	9 (75%)	0.43
Prior CABG, n (%)		11 (91.7%)	9 (75%)	0.59
One-vessel disease, n (%)		0	2 (16.7%)	0.22
Two-vessel disease, n (%)		3 (25%)	2 (16.7%)	1.00
Three-vessel disease, n (%)		9 (75%)	8 (66.7%)	0.67
Chronic total occlusion, n (%)		10 (83.3%)	9 (75%)	1.00
Ejection fraction (EF) (%)		58 ± 6	58 ± 9	0.57
End diastolic volume indexed (EDVi)		66 ± 12	69 ± 22	0.18
Ischemia location				
	Anterior	5 (41.7%)	7 (58.3%)	0.41
	Anterolateral	6 (50%)	5 (41.7%)	0.68
	Inferolateral	7 (58.3%)	5 (41.7%)	0.41
	Inferior	3 (25%)	2 (16.7%)	0.62
	Septal	3 (25%)	1 (8.3%)	0.27
	Reversible perfusion defect (%)	8.9 ± 7.1	8.8 ± 6.1	0.97
	Fixed perfusion defect (%)	12 ± 6.3	9.7 ± 6.3	0.41
Antiarrhythmic therapy				
	Beta blocker, n (%)	12 (100%)	12 (100%)	1.00
	Ivabradine, n (%)	2 (16.7%)	0	0.48
	Ranolazin, n (%)	10 (83.3%)	12 (100%)	0.48
	CSR occlusion pressure–mmHg ± SD	56 ± 10	/	

CABG, coronary artery bypass grafting; Sham, a sham procedure group; CSR, coronary sinus reducer group; PCI, percutaneous coronary intervention; Sham, sham procedure group; SD, standard deviation.

**Fig. 2.**
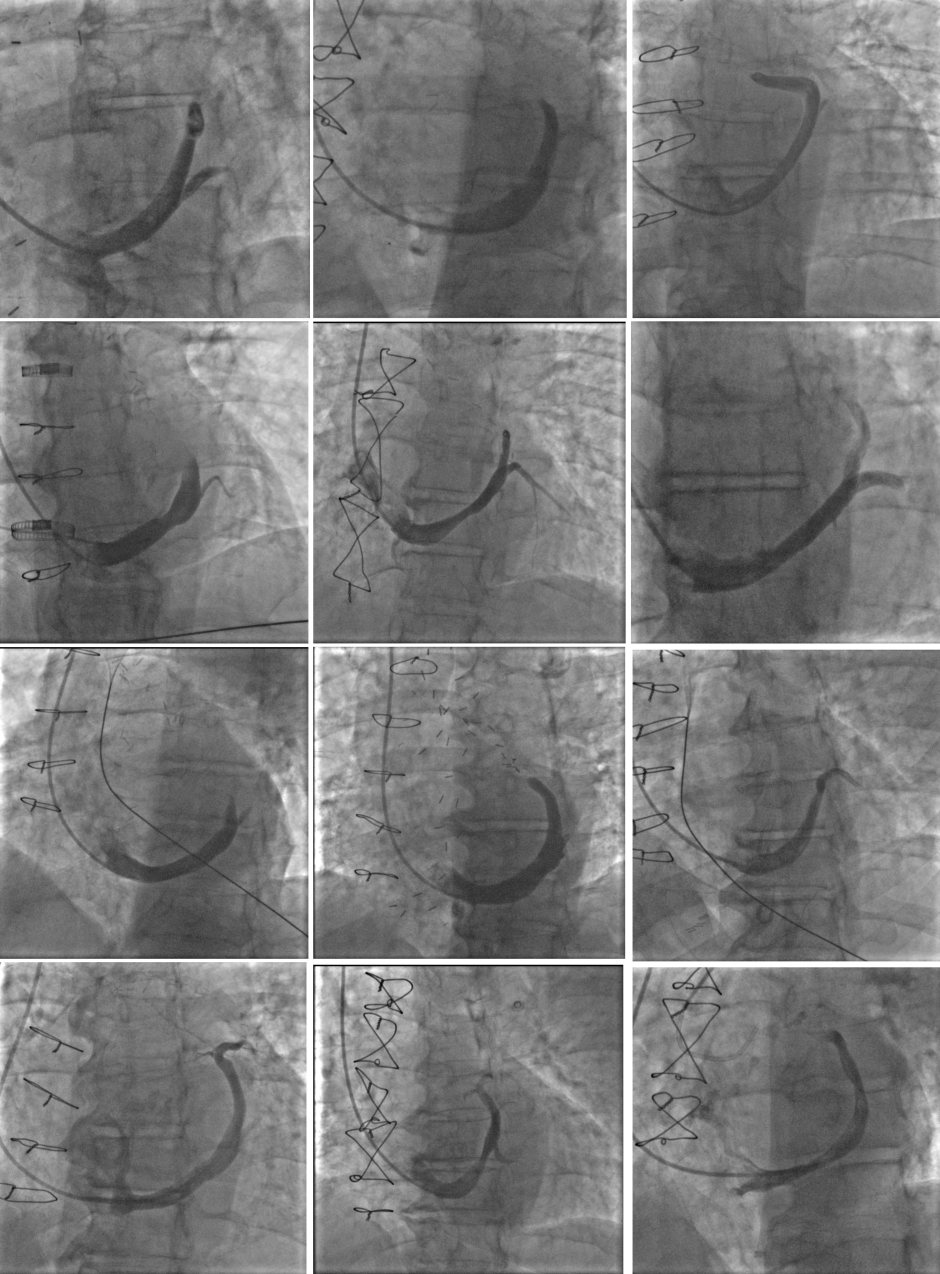
**Coronary sinus venograms of 12 patients receiving coronary sinus reducer**.

Most patients were male with extensive coronary artery disease. Altogether, 63% of patients underwent previous percutaneous, and 83% underwent previous surgical, revascularization. 83% of patients in the CSR group and 75% in the sham group had a non-revascularized chronic total occlusion (CTO) of at least one coronary artery. The extent of reversible ischemia was comparable between both groups and was primarily confined to the territory of the left coronary artery. All patients were receiving beta-blockers, and 92% of patients were receiving ranolazine. CSR implantation was successful in all patients randomized to the CSR group. Intraprocedural venograms of patients receiving CSR are presented in Fig. 2. Vein tributaries were delineated and allowed distal CSR implantation without visible lateral vein distal to the CSR narrowing. Inferior heart veins were drained to the distal end of the coronary sinus or separately to the right atrium.

The mean heart rate at baseline was 66 ± 11 bpm in CSR and 61 ± 11 bpm in the sham group and did not change at follow-up (*p* = 0.96 and 0.20, respectively) (Table 2). SDNN was also comparable between both groups (*p* = 0.41) and did not change at follow-up (*p* = 0.86 and *p* = 0.20, respectively). Low, high, and total frequency powers at baseline did not differ and remained unchanged at follow-up. QRS-T angles using peak and mean amplitudes did not differ at baseline or change after the procedure. TpTe values were comparable at baseline (*p* = 0.70), and the change after the procedure was insignificant. SVG values were also comparable at both baseline and follow-up. QT variability parameters also remained unaffected.

**Table 2.** **Repolarization parameters before and after the procedure**.

		CSR (n = 12)		Sham (n = 12)		* $p^{†}$ *
Baseline						
	Heart rate (bpm)	66 ± 11		61 ± 11		0.25
	SDNN	24.4 (16.6–39.9)		22.8 (22.1–27.4)		0.41
	HRVlf	4.61 ± 1.51		3.97 ± 0.9		0.25
	HRVhf	4.23 ± 2.44		3.4 ± 1.23		0.32
	HRVtot	5.0 (4.74–6.29)		5.2 (5.09–5.8)		0.70
	QRSTP	71.9 ± 33.4		65.7 ± 26		0.63
	QRSTM	74.4 ± 34.2		67.1 ± 24.8		0.57
	TpTe	92.0 (87.0–97.0)		91.3 ± 9.2		0.70
	SVG	54.3 ± 20.1		51.9 ± 14.5		0.76
	VR	0.71 ± 0.61		1.0 ± 0.56		0.53
	QTVi	0.24 ± 0.20		0.16 ± 0.11		0.60
6 months			p*		p*	*p*º
	Heart rate (bpm)	66 ± 10	0.96	64 ± 9	0.20	0.37
	SDNN	24.8 (15.4–47.7)	0.86	31.3 (21.2–45.5)	0.48	0.60
	HRVlf	5.02 ± 1.75	0.31	4.4 ± 1.34	0.41	1.00
	HRVhf	5.57 ± 2.38	0.08	3.93 ± 1.88	0.47	0.42
	HRVtot	6.61 (4.53–8.45)	0.18	5.71 (5.09–6.12)	0.48	0.79
	QRSTP	74.6 ± 34.7	0.52	71.4 ± 34.2	0.29	0.54
	QRSTM	75.1 ± 34.2	0.83	71.9 ± 34.6	0.38	0.47
	TpTe	84.0 (75.0–94.0)	0.20	95.3 ± 19.7	0.24	0.11
	SVG	47.5 ± 15.8	0.06	54.2 ± 14.9	0.97	0.12
	VR	0.81 ± 0.69	0.62	1.17 ± 0.62	0.47	0.46
	QTVi	0.37 ± 0.10	0.20	0.18 ± 0.12	0.58	0.62

CSR, coronary sinus reducer group; Sham, a sham procedure group; bpm, beats per minute; SDNN, standard deviation of the normal-to-normal intervals; QRST, the spatial angle between QRS and T axis using mean (QRSTM) and peak (QRSTP) values; TpTe, T peak and T end interval; SVG, spatial ventricular gradient; VR, QT variability ratio; QTVi, QT variability index; HRVlf, low-frequency power of heart rate variability; HRVhf, high-frequency power of heart rate variability; HRVtot, total power of heart rate variability; *, *p* for intragroup comparison at baseline and at follow-up; ^†^, *p* for intergroup comparison at baseline; º, *p* for intergroup comparison of change between baseline and follow-up.

## 4. Discussion

To the best of our knowledge, this is the first study exploring the arrhythmic effects of CSR implantation in patients with refractory angina pectoris. CSR implantation did not significantly impact the arrhythmogenic substrate compared to the sham procedure.

Recently reported results of the Crossroad study showed improved aerobic exercise capacity with increased oxygen consumption after CSR implantation, which was in line with the Cosira trial, which showed an improvement in CCS angina score and quality of life [12, 13]. Both studies were randomized and blinded with a sham procedure. Some non-randomized studies also showed the improvement of left ventricular perfusion by SPECT or magnetic resonance imaging [14, 15, 16, 17]. However, potential antiarrhythmic effects were not assessed.

The rationale for this study was the striking results from the study series conducted by Kralios *et al*. [5], which demonstrated a linear increase in ventricular fibrillation threshold (up to 82%) with an increase of the coronary sinus pressure up to 41.2 ± 1.4 mmHg. This increase in fibrillation threshold was achieved in normally perfused hearts without induced ischemia. In another study, coronary sinus obstruction delayed or prevented the occurrence of ventricular fibrillation and reduced ventricular ectopy in hearts with induced ischemia in the territories of two coronary arteries [6]. Prevention of fibrillation was again positively correlated with coronary sinus pressure. Although these results cannot be directly translated to human hearts with ischemic heart disease treated with CSR implantation, the pathophysiology of these patients may be the closest pathophysiological approximation of these preclinical studies.

As suggested previously, sinus pressure may be the primary predictor of antiarrhythmic effects. Coronary sinus pressure after CSR implantation highly depends on the extent of Thebesian veins and consequent drainage of venous blood to the ventricles bypassing the coronary sinus. Extensive drainage through Thebesian veins was already reported as a possible mechanism of inadequate antianginal efficacy of CSR [18]. To limit the influence of this phenomenon and avoid possible heterogeneity in the study group we prospectively measured the occlusion pressure during CSR implantation. The mean systolic occlusion pressure in patients with implanted CSR was 56 ± 10 mmHg, and the lowest pressure was 45 mmHg, excluding the antagonistic effect of the extensive Thebesian network.

Arrhythmia is a frequent and life-threatening complication of ischemic heart disease with an incidence of 2–4% [19]. There is an essential difference between the arrhythmogenic substrate of acute and chronic ischemia. Ventricular arrhythmias in acute ischemia are the result of abnormal automaticity, triggered activity, and micro reentry due to transmural voltage gradients [20]. In contrast, monomorphic ventricular tachycardia encountered in the chronic phase of the disease results from reentry circuits associated with scar areas [21]. This difference in arrhythmogenic substrate might mitigate the effect of coronary sinus pressure augmentation in chronic ischemic heart disease as there is a less direct relation between the occurrence of reentry arrhythmias and the homogeneity of extracellular environment achieved by the preservation of the normal microvascular pressure [21, 22]. While all our patients had demonstrable reversible ischemia by SPECT, the hrECG was recorded at rest, which may have underestimated the arrhythmogenic potential of ischemia during exercise.

While the risk for ventricular tachycardia is high in the acute phase of the ischemic disease, it tends to decline over time [23]. However, the incidence of ventricular arrhythmias is higher in patients with more extensive scars and more advanced ventricular dysfunction [20, 23]. While more than 80% of our patients had non-revascularized chronic total occlusion, they did not have symptomatic heart failure and had a preserved ejection fraction. As the rate of major cardiac events in patients with refractory angina is relatively low, the main aim of therapy remains the improved quality of life [24]. The antianginal effects of CSR therapy in chronic ischemia differ from the antiarrhythmic effects of coronary sinus pressure augmentation demonstrated in acute ischemia, as sinus obstruction during the acutely induced ischemia did not affect regional perfusion nor improved the collateral blood flow, which has been demonstrated in the chronic phase [6]. While preclinical data suggest antiarrhythmic benefits, further research is needed to understand the intricate relationship between CSR implantation, myocardial perfusion, and arrhythmogenesis.

### Study Limitations

A relatively small number of included patients due to the single-center design and a limited number of patients eligible for this treatment limits the strength of our findings. The study included eligible patients enrolled in the Crossroad study. Due to scarce data in the literature, prior calculation of the sample size was not possible. As it was a clinical study, the assessment of arrhythmic properties was limited to noninvasive analysis of hrECG parameters at rest, which may have underestimated the arrhythmic changes that might be evident with invasive testing or during exercise. While we measured the occlusion pressure during CSR implantation, we could not correlate ECG parameters to coronary sinus pressure during the recording.

## 5. Conclusions

Compared to the sham procedure, CSR implantation did not significantly impact the arrhythmogenic substrate assessed with hrECG. The results are in contrast to the preclinical data reporting the beneficial effects of coronary sinus pressure augmentation on the occurrence of ventricular arrhythmias.

## Data Availability

The data presented in this study are available upon request from the corresponding author and are not publicly available due to ethical issues.
